# Adventures in data citation: sorghum genome data exemplifies the new gold standard

**DOI:** 10.1186/1756-0500-5-223

**Published:** 2012-07-02

**Authors:** Scott C Edmunds, Tom J Pollard, Brian Hole, Alexandra T Basford

**Affiliations:** 1 GigaScience, BGI-Hong Kong Ltd., 16 Dai Fu Street, Tai Po Industrial Estate, NT, Hong Kong; 2 Ubiquity Press, Gordon House, 29 Gordon Square, London, WC1H 0PP, United Kingdom; 3 Department of Space & Climate Physics, Mullard Space Science Laboratory, University College London, Surrey, RH5 6NT, United Kingdom; 4 University College Hospital, 235 Euston Road, London, NW1 2BU, United Kingdom; 5 Institute of Archeology, University College London, 31–34 Gordon Square, London, WC1H 0PY, United Kingdom

## Abstract

Scientific progress is driven by the availability of information, which makes it essential that data be broadly, easily and rapidly accessible to researchers in every field. In addition to being good scientific practice, provision of supporting data in a convenient way increases experimental transparency and improves research efficiency by reducing unnecessary duplication of experiments. There are, however, serious constraints that limit extensive data dissemination. One such constraint is that, despite providing a major foundation of data to the advantage of entire community, data producers rarely receive the credit they deserve for the substantial amount of time and effort they spend creating these resources. In this regard, a formal system that provides recognition for data producers would serve to incentivize them to share more of their data.

The process of data citation, in which the data themselves are cited and referenced in journal articles as persistently identifiable bibliographic entities, is a potential way to properly acknowledge data output. The recent publication of several sorghum genomes in *Genome Biology* is a notable first example of good data citation practice in the field of genomics and demonstrates the practicalities and formatting required for doing so. It also illustrates how effective use of persistent identifiers can augment the submission of data to the current standard scientific repositories.

## Discussion

One of the key lessons learned from the Human Genome Project, taking a page from the *C. elegans* community [1], was that making data broadly and freely available prior to publication was profoundly valuable to the field of genomics [2]. Subsequent genomics projects have tried to follow this practice as laid out in the Bermuda Rules [3] and ultimately enshrined in the Fort Lauderdale agreement [4]. The wider biological science community has also attempted to follow similar practices, as outlined in the guidelines published from the Toronto International Data Release Workshop [5], but adoption has been held back by a lack of easy-to-access repository infrastructure for many fields as well as an absence of incentives for authors to go through the time and effort necessary to make their work openly and easily available to others.

The benefits of making data available to the research community as a whole can be calculated [6]: there is a measurable trend towards an author’s work accumulating additional citations as a result of the supporting data being publically accessible [7,8]. Again, however, the lack of a universally recognized tagging system linking investigators to their deposited data has hindered authors from receiving due credit [9]. Recent scandals relating to falsified data that went long undetected in medicine [10] and psychology [11] also highlight the need to make data easily accessible for purposes of validation and to maintain public trust in science. One notable attempt to address the issues of gaining complete access to data is the Dryad repository, which serves as a storehouse for smaller datasets directly affiliated with publications in the biosciences [12].

The next step forward in this regard is the recent publication of the genomes of three strains of the important food crop *Sorghum bicolor* published in *Genome Biology*[13]. This work follows the best practices of the genomics community by having the supporting raw and useful processed data available in the relevant and available data repositories. However, for the first time in the long-established data-sharing practices of the community, this process has been supplemented specifically by integrating into the reference section a citation for the collective dataset. Thus, in addition to having the raw data [SRA046843], assemblies [GenBank: AHAO00000000-AHAQ00000000], mutation [1056306], and structural variation data [nstd63] available in a number of NCBI databases, the data citation included in the reference section [14] makes available the same data, along with additional information, from a single point of access. In addition, and perhaps of even greater value, these data are persistently linked via a citable DataCite Digital Object Identifier (DOI), and are hosted on the *GigaScience*[15]*Giga*DB database [16].

## *GigaScience* and *Giga*DB

*GigaScience* is a journal and data publishing project set up in conjunction with BGI (formally the Beijing Genomics Institute) [17], one of the world’s largest genomics data producers, and BioMed Central [18]. Using BGI’s large data storage and cloud computing infrastructure, *GigaScience* has created a novel publication format that integrates manuscript publication with data hosting. The data associated with articles are hosted in the connected *Giga*DB database and given DOIs to make them more searchable and trackable as well as independently citable.

To demonstrate the utility of data citation and to promote extremely rapid data release and dissemination of unpublished datasets independently of the journal, *Giga*DB has recently released large-scale data resources created by the BGI and its many external collaborations. Importantly, these data are the first cases in which whole genome-type data have been released with DOIs. *GigaScience* has been working with DataCite through the British Library to enable these and future datasets to receive DOIs.

## DataCite

Founded in December 2009, DataCite is an international partnership working towards a global citation framework for research data, with the aim of enabling researchers to find, access, and reuse datasets with confidence. Since its foundation, DataCite has been building a community to collaboratively develop services and good practices for data citation [19,20].

DataCite is initially leveraging the DOI system for research data. DOIs identify a resource, rather than the location of the resource, allowing the creation of persistent and stable references and offering an easy way to connect articles with their underlying data. The DOI system is governed by the International DOI Foundation (IDF), a non-profit organisation, with DataCite operating as a Registration Agency. This relationship confers DataCite and its member organisations [21] with rights and infrastructure to register DOIs. The crucial advantage of the DOI system over alternatives is that it is already familiar to researchers, publishers, and libraries.

DataCite provides a service for trusted repositories to mint DOIs for datasets and, since August 2011, collects mandatory metadata on every DOI minted. Services are being developed around this open ‘metadata store’ to promote discovery and to facilitate access to original cited datasets. DataCite DOIs resolve to public pages describing the datasets and providing a route for access. DataCite is also developing a content negotiation service that will allow metadata, and possibly even the datasets themselves, to be requested directly via DOIs [22].

A cost is associated with managing persistence and with assigning identifiers, and so there is a cost associated with DOIs. In April 2011 the International DOI Foundation changed the charging model from a per-DOI cost to a cost-sharing model [23]. Essentially this means that repositories can pay a flat-fee to a Registration Agency for the right to mint virtually unlimited DOIs.

## A brief history of data citation

Environmental sciences researchers have been using the Pangaea [24] database to host data associated with their manuscripts for many years (for an example from 2005 see [25]), and Dryad has more recently created a similar model with biomedical data [26]. These have been notable successes in the movement for better data access. However, in cases where journals have included such datasets in their references, the datasets are often treated and formatted the same way as links on the web and, thus, are not listed by citation indices (such as article [25] and its associated dataset [27]). The long-established Protein Data Bank (PDB) biological macromolecular structure data archive [28] also uses DOIs, but other than rare exceptions [29,30], very few research articles have used DOIs to reference structures. Publishing data by wrapping and integrating it into the established journal infrastructure is underway in a number of research areas such as the *Earth Systems Science Data* journal [31], and there have been attempts to semantically enhance and integrate links with data via DOIs in biodiversity [32] and infectious disease research [33]. This has facilitated discovery and access to the underlying data, but these examples have not utilized or been citable using current journal indexing services.

As DOIs issued for *Giga*DB datasets have been associated with and published alongside journal manuscripts, the *Giga*DB project appears to be the first time that genomic datasets have been released prior to manuscript publication in this citable DOI form. Although there have been public calls [2] and journal editorials [9] encouraging such a system, the practicalities and consequences of releasing data in a citable form before the publication of their associated manuscripts have been unclear, especially with widely varying journal editorial policies regarding pre-publication dissemination of results. Relevant to this is a commonly acknowledged editorial guideline from the *New England Journal of Medicine* that outlines limitations on prepublication release of information known as the “Ingelfinger rule” [34]. It effectively states that a manuscript may not be considered for publication if its substance has been submitted or reported elsewhere and it has made many researchers wary of publicizing preliminary data. However, there are a number of ambiguities as to how this restriction is reconciled with the biological, and in particular the genomic, community’s code of practice regarding pre-publication data deposition in public databases.

An interesting test case was the first dataset to be given a data citation by BGI [35]. This high-profile dataset, the first publicly available genome of the *E. coli* 0104:H4 pathogen responsible for the 2011 European outbreak, was released prior to the publication of an associated article [36]. Researchers at the BGI collaborated with the University Medical Centre Hamburg-Eppendorf to rapidly sequence the genome of the pathogen. Due to the seriousness of the situation (with 50 human deaths and over 4000 people infected), it was clear that it was not in the public’s best interest to hold back that information in order to follow standard research practices of first analysing the data and then waiting to release them until after acceptance of the resulting manuscript. Instead, the decision was made to immediately release the dataset under the most open public domain waiver, CC0 [37], to maximize its use by the community. By giving the dataset a DOI it was possible to not only enable the research community to cite and credit the authors, but also to mark the time of data release, making this less commonly used route of data release more attractive to the authors.

Of greatest interest, perhaps, was that the rapid release of the *E. coli* genome data enabled an international community of “crowdsourced” researchers to pool resources and carry out expeditious “open-source” analysis of the organism, a level of instantaneous collaboration that has not been seen before. This high-profile distributed problem-solving approach substantially aided in limiting the health crisis, with strain-specific diagnostic primers disseminated within five days of the release of the sequence data (sequence available from the DOI landing page [35]), and the draft unassembled genome sequence data subsequently enabled the development of a targeted bactericidal agent to kill the pathogen [38]. It also brought to light a potentially useful way of scientifically addressing similar outbreaks in the future. Additionally, results of these analyses were published in the *New England Journal of Medicine* a few months later, showing that data citation can complement the traditional forms of academic credit [36].

Many other unpublished datasets have been released in the *Giga*DB database with DataCite DOIs, and a number of these have subsequently been used in scholarly journal articles. DOIs for two BGI datasets [39,40] used in a *Nature Biotechnology*[41] article were listed in the article’s Accession Codes section, but were not included in the reference section due to citation limits and policies treating them as non-refereed sources of information such as websites. The authors of the *Sorghum bicolor* genome paper worked very closely with the editors of *Genome Biology* to ensure that it followed the best practice guidelines and current recommendations regarding how best to cite data, and included the data citation in the reference section [42]. Since the publication of this paper, there have been positive recent developments from publishers such as Springer and Nature providing examples of data cited in this way [43,44].

## Discoverability, accessibility, and preservation

Discoverability and accessibility of data are separate issues and should be treated as such. There are times when it may be necessary to limit accessibility to a dataset, but this should not prevent it from being archived and made discoverable via open metadata and a persistent identifier such as a DOI. Equally, discoverability and long-term preservation of data can be dealt with as separate issues. Repositories should be carefully selected to ensure a preservation plan is in place, but we must accept that some datasets will be lost over time, for example due to limited storage capacity. By ensuring that persistent identifier organizations are provided with open metadata, it should at least be possible to keep a record of a dataset’s existence and provenance however. DataCite, for example, collects metadata for all datasets that are allocated DataCite DOIs. In the event of a dataset becoming unavailable, the appropriate DOI can be updated to resolve to the associated metadata record.

## How to cite data

Given the importance of data in promoting research, and the needs of data producers to gain credit for their work in the same manner that researchers using these data are recognized, datasets should be cited as research articles are cited. Further to this, even though many journals currently tend to remove URLs from the reference list, both DataCite and CrossRef recommend displaying DOIs within references as full URLS. They consider this best practice because it emphasises the actionable link and allows readers to readily access the underlying data. The DOI in URL form not only serves the same function as a journal volume, issue and page number do for a printed article, but also gives the combined advantages of linked access and the assurance of persistence, the lack of which in the past being part of the reason many journals have been reluctant to cite plain vanilla URLs. An example of what can be considered a new gold standard for data citation is the way in which the data that underpin the recently published sorghum paper [14] were cited in the reference section, as follows:

Zheng, L-Y; Guo, X-S; He, B; Sun, L-J; Peng, Y; Dong, S-S; Liu, T-F; Jiang, S; Ramachandran, S; Liu, C-M; Jing, H-C (2011): Genome data from sweet and grain sorghum (Sorghum bicolor). *GigaScience*. http://dx.doi.org/10.5524/100012

It is important to note that the DOI will always point to the version of the data that were used for the study in which they were cited, enabling other researchers to use them for validation and comparative studies with confidence.

Formally citing the dataset in this manner not only clearly identifies the dataset, but also paves the way for data discovery and citation tracking via existing bibliometric services. These services have traditionally focused on DOIs in the reference section only, so including data citations here can help such services to start using them more quickly as existing systems will require less modification.

## Aiding the adoption of data citation

Many journals now include ‘Data Accessibility’ sections that provide information about the data used in the paper and explain where these data can be accessed. Journal editors are also starting to draw up new guidelines for data citation, but these approaches still remain inconsistent. In this regard, it is crucial that high-profile journals take the lead in citing data in a manner that drives adoption of good practices and raises awareness of this issue to the broader research community.

Journal editors and publishers who promote consistent and equitable means of citing data, as exemplified by the handlers of the sorghum paper [13], should be commended. Defining formal mechanisms for dataset citation is essential for making datasets more readily tracked and easily accessed. Furthermore, it provides the only real means for data producers to obtain appropriate recognition for their work, promoting more rapid data release potentially prior to the much more time-consuming process of manuscript publication. It also gives recognition and makes clear the role of the researchers investing the most effort in producing the dataset, who may not have received similar credit in an eventual more analysis-focused publication. As research is being carried out with ever increasing amounts of data, widespread data availability will serve to enhance scientific progress and provide greater public benefit from the investments made to create these sharable data resources.

## Competing interests

SCE and AB are employees of the BGI and work on the *GigaScience* project. TP is a previous employee of the British Library and DataCite. BH is a previous employee of the British Library and Dryad-UK.

## Authors' contributions

SE, TP and AB assigned DOIs to the BGI datasets highlighted here. All authors contributed to the writing, and read and approved the final manuscript.
